# Gene Expression in Peripheral Blood Leukocytes in Monozygotic Twins Discordant for Chronic Fatigue: No Evidence of a Biomarker

**DOI:** 10.1371/journal.pone.0005805

**Published:** 2009-06-05

**Authors:** Andrea Byrnes, Andreas Jacks, Karin Dahlman-Wright, Birgitta Evengard, Fred A. Wright, Nancy L. Pedersen, Patrick F. Sullivan

**Affiliations:** 1 Department of Genetics, University of North Carolina at Chapel Hill, Chapel Hill, North Carolina, United States of America; 2 Department of Biostatistics, University of North Carolina at Chapel Hill, Chapel Hill, North Carolina, United States of America; 3 Department of Medical Epidemiology and Biostatistics, Karolinska Institutet, Stockholm, Sweden; 4 Department of Biosciences and Nutrition, Karolinska Institutet, Stockholm, Sweden; 5 Department of Clinical Microbiology, University of Umeå, Umeå, Sweden; Université de Toulouse, France

## Abstract

**Background:**

Chronic fatiguing illness remains a poorly understood syndrome of unknown pathogenesis. We attempted to identify biomarkers for chronic fatiguing illness using microarrays to query the transcriptome in peripheral blood leukocytes.

**Methods:**

Cases were 44 individuals who were clinically evaluated and found to meet standard international criteria for chronic fatigue syndrome or idiopathic chronic fatigue, and controls were their monozygotic co-twins who were clinically evaluated and never had even one month of impairing fatigue. Biological sampling conditions were standardized and RNA stabilizing media were used. These methodological features provide rigorous control for bias resulting from case-control mismatched ancestry and experimental error. Individual gene expression profiles were assessed using Affymetrix Human Genome U133 Plus 2.0 arrays.

**Findings:**

There were no significant differences in gene expression for any transcript.

**Conclusions:**

Contrary to our expectations, we were unable to identify a biomarker for chronic fatiguing illness in the transcriptome of peripheral blood leukocytes suggesting that positive findings in prior studies may have resulted from experimental bias.

## Introduction

The etiology of chronic fatigue syndrome (CFS) is unknown [1]–[3]. Many theories of the pathophysiology of CFS have been suggested [4]–[9], often based on suspicions of the role of an acute viral illness or immune dysfunction. The availability of a biomarker for CFS would be of particular benefit for clinical and basic research.

Gene expression studies of peripheral blood leukocytes (PBLs) are a potentially promising source of biomarkers for CFS [10]. PBLs are both accessible and salient for CFS given the prominence of immune and infectious theories of its etiology. Moreover, gene expression patterns in human PBLs are not unrelated to less accessible tissues like brain [11]. We are aware of four published non-overlapping studies that compared gene expression in PBLs in cases with CFS in comparison to controls [12]–[15]. These small studies included a total of only 45 cases (5, 7, 8, and 25) and, of the 108 transcripts reported to have altered expression, only one transcript was altered in more than one study (*MSN*, moesin). [13], [15]

Given the lack of clarity in existing studies of CFS, we undertook an “unbiased”, transcriptome-wide search for gene expression changes associated with CFS in PBLs. Our study had two notable design features. First, we used a control group optimal for detecting state-related gene expression changes and minimized false-positive findings due to genetic mismatching between cases and controls as we contrasted 44 individuals with clinically-evaluated chronic fatiguing illness with their 44 unaffected monozygotic co-twins. Use of rigorously discordant monozygotic twins provides the best control for genetic background currently possible in humans and allows use of paired statistics with greater statistical power. Second, we carefully standardized sampling conditions so that PBL samples were drawn into RNA-stabilizing media and taken from both members of a twin pair at the same time and place.

## Materials and Methods

### Ethics Statement

The protocol was approved in advance by the ethical review board at UNC-CH and the Karolinska Institutet and all subjects provided written informed consent.

We screened ∼61,000 individual twins from the Swedish Twin Registry for the symptoms of fatiguing illness [16]–[18]. All twins were born in Sweden of Scandinavian ancestry. Of 5,597 monozygotic twin pairs where both were alive and had provided usable responses to CFS screening questions, we identified 140 pairs of twins who met preliminary inclusion criteria: born 1935–1985, classified as a monozygotic twin based on questionnaire responses [19], and discordant for chronic fatiguing illness (i.e., one twin reported substantial fatigue and the other twin was evidently well). A telephone interview using a standardized script was used to assess eligibility for participation. Twins who remained eligible attended a half-day clinical assessment by a specially trained physician at the Karolinska Institutet in Stockholm. At this visit, a CFS-focused medical assessment was conducted that included standardized medical history, physical examination, and screening biochemical, hormonal, and hematological studies in accordance with international recommendations [1].

Of 140 monozygotic and preliminarily discordant twin pairs, one or both twins declined participation in 23 pairs, 25 pairs were concordant for CFS-like illness, and inclusion criteria were not met in 35 pairs (e.g., chronic fatigue had resolved or an illness that could explain fatiguing symptoms such as neoplasia had emerged). After excluding these 83 pairs, 57 pairs of twins attended the clinical evaluation sessions, and 10 pairs were found not to meet inclusion criteria (9 pairs were concordant for the presence or absence of chronic fatigue or a medical explanation was detected – e.g., newly diagnosed type 2 diabetes mellitus – and 1 pair was dizygotic). Zygosity was confirmed by genotyping 46 single nucleotide polymorphisms using two Sequenom iPlex panels. In 3 pairs, microarray data of satisfactory quality could not be obtained.

The analysis sample consisted of 44 pairs of rigorously discordant and genetically proven monozygotic twins. Discordance was defined as one twin meeting criteria for either idiopathic chronic fatigue (ICF, 12 pairs) or CFS (32 pairs) [1], [2] and the co-twin was required never to have experienced impairing unusual fatigue or tiredness lasting more than one month. Thus, all affected twins were required to have current, long-standing (≥6 months), medically unexplained fatigue associated with substantial impairment in social and occupational functioning and the unaffected co-twins were effectively well. A diagnosis of CFS adds a requirement for ≥4 of 8 specific symptoms (e.g., unrefreshing sleep, muscle pain) to that of ICF. We explain elsewhere the rationale for including ICF along with CFS based on phenotypic [17] and twin analyses [18].

Transient/situational factors can influence gene expression measurements. Biological sampling was standardized by having samples drawn from both members of a twin pair at the same place and time (∼0900) after an overnight fast. We required that all subjects be in their usual state of health on the day of sampling (i.e., no acute illness or recent exacerbation of a chronic illness). It was neither practical nor ethical to study subjects medication-free, but we delayed assessment if there had been a recent significant dosage change.

Peripheral venous blood was drawn using sterile technique into PAXgene tubes manufactured in the same batch (Qiagen, to protect RNA from degradation and to minimize *ex vivo* gene expression). Total RNA was purified using the PAXgene blood RNA kit following the manufacturer's instructions (Qiagen). RNA quality was determined using the Agilent 2100 Bioanalyzer. Total RNA (5.0 µg) was labeled with the one-cycle cDNA synthesis kit (Invitrogen) and spiked with eukaryotic Poly-A RNA controls to check the target labeling process (Affymetrix). Synthesized cDNA was transcribed *in vitro* using the GeneChip IVT labeling kit (Affymetrix). The biotin labeled cRNA product (20 µg) was purified with a sample cleanup module (Qiagen) and samples were fragmented with the fragmentation buffer from Affymetrix at 94°C for 35 minutes. Fragmented and labeled targets (together with hybridization and oligo B2 controls) were hybridized to Affymetrix Human Genome U133 Plus 2.0 arrays at 45°C for 16 hours. Washing and staining of the arrays were performed on the Affymetrix fluidics station using the EukGE-WS2v5_450 protocol. Imaging of the arrays and signal quantification were performed with the Affymetrix GeneChip Scanner 3000 and GeneChip Operating Software. For verification, we used qRT-PCR. RNA was converted to cDNA with Superscript III (Invitrogen) and qRT-PCR was run with ABI's Taqman gene expression assays (with 18S rRNA as control). The ΔΔCt method was used for the calculations.

Array images were manually checked for defects using DChip [20], [21] and then normalized using the RMA algorithm in Affymetrix Expression Console (v1.0). After normalization, the Bioconductor [22] Significance Analysis of Microarrays package [23] was used to compute modified paired t-tests that contrasted an affected twin with the unaffected co-twin for each transcript using R [24]. To adjust for multiple comparisons, the nominal permutation-based p-values from SAM were used to compute false discovery rate q-values [25]–[27]. Pathway analyses for KEGG pathways [28], GO keywords [29] (biological process, cellular component, and molecular function), and PFAM protein family groupings [30] were conducted using SAFE which performs array permutation to account for transcript correlation [31], [32]. These expression data are available from GEO (http://www.ncbi.nlm.nih.gov/geo) under accession number GSE16059 and were prepared in accordance with MIAME 2.0 standards.

## Results

The analytic data set consisted of microarray data from 44 pairs of monozygotic twins discordant for clinically-evaluated chronic fatiguing illness (***Table 1***). Most pairs were female (89%), and the median age at evaluation was 51 years. Of the affected twins, 32 met criteria for CFS and 12 for ICF with a median duration of chronic fatigue of 8 years with no significant difference between affected twins with CFS and ICF (paired t_43_ = 0.32, p = 0.75). Body mass index was similar between the affected and unaffected twins. Two affected individuals (4.5%) reported sudden onset of fatigue. Affected twins had significantly worse physical and mental functioning on the SF-36 [33] and reported significantly greater current fatigue. The mean functioning of affected twins was over a standard deviation worse than Swedish norms whereas the unaffected twins were similar to Swedish norms (http://www.sf-36.org/nbscalc/index.shtml, accessed 12 December 2008).

**Table 1:** Descriptive characteristics for 44 pairs of monozygotic twins discordant for chronic fatiguing illness.

Variable	Affected twins	Unaffected co-twins	Statistical comparison
Met criteria for CFS	32/44, 73%	0/44, 0%	*By design*
Met criteria for ICF	12/44, 27%	0/44, 0%	*By design*
Female sex	39/44, 89%	39/44, 89%	*Identical by design*
Median age at evaluation, IQR	51, 39–59 years	51, 39–59 years	*Identical by design*
Median body mass index, IQR	24.6, 22–30 kg/m^2^	24.4, 22–31 kg/m^2^	Paired t_43_ = 0.2, p = 0.87
Median SF-36 physical function, IQR	41.2, 28–48	48.2, 40–52	Paired t_43_ = 3.0, p = 0.004
Median SF-36 mental function, IQR	38.9, 29–47	51.6, 40–56	Paired t_43_ = 5.0, p = 1×10^−5^
Median current fatigue by VAS, IQR	69, 49–77	19, 10–51	Paired t_43_ = −7.2, p = 6×10^−9^
Median duration of fatigue, IQR	7.6, 4–16 years	NA	–
Sudden onset of fatigue	2/44, 4.5%	NA	–

Abbreviations. CFS = chronic fatigue syndrome, ICF = idiopathic chronic fatigue, IQR = inter-quartile range, VAS = visual analog scale (0–100, higher means greater fatigue at clinical examination).

The main analyses contrasted gene expression in PBLs in 44 pairs of monozygotic twins affected with CFS or ICF to that of their unaffected co-twins. As inclusion of males could increase noise, the second planned analysis compared 39 female pairs with CFS or ICF and the third planned analysis compared the 28 pairs of female twins with CFS. For each of these three sets of statistical comparisons, the observed results did not deviate from those expected by chance (***Figure S1***). When we compared our findings to a list of 108 transcripts reported as differentially expressed in CFS [12]–[15], 107 were studied in our experiment, 101/107 had p>0.1 in our study, and only two had p<0.05 (*CXCR4* p = 0.03 and *RAP2C* p = 0.04), a degree of overlap that does not depart from chance expectations. At the single transcript level, there was no biological evidence of altered gene expression in PBLs that correlated with chronic, impairing, and medically unexplained fatiguing illness. For verification, we used qRT-PCR to assess the expression of seven genes selected from the CFS gene expression literature and our empirical findings (*ANKLE2*, *BLKE*, *BRD1*, *CPA3*, *DCTN1*, *ICAM*, and *ORC*). All p-values from paired t-tests contrasting affected and unaffected monozygotic twins were ≥0.26.

It is possible that functionally-related genes might have important set-wise gene expression changes with no individual transcript meeting criteria for significance. As a hypothesis-generating analysis, we used SAFE [31] to conduct analyses of gene groupings defined by KEGG pathways [28], GO keywords [29] (biological process, cellular component, and molecular function), and PFAM protein family groupings [30]. Broadly, these analyses revealed significant differences in cell replication processes and amino acid and lipid metabolic pathways (***Table S1***). These results do not map directly onto current major theories of CFS pathogenesis and should be regarded as hypothesis-generating.

## Discussion

### Main Finding

The overarching goal in this study was to attempt to identify one or more biomarkers for chronic fatiguing illness via a comprehensive search of the “transcriptome” in an accessible tissue (peripheral blood leukocytes, PBLs) plausibly involved in the pathophysiology of this idiopathic syndrome. We attempted to correct methodological issues in prior reports by careful control of sources of bias (e.g., by studying discordant monozygotic twin pairs, use of RNA stabilizing media, and standardized sampling conditions). We found no evidence of differential PBL gene expression that characterized the presence or absence of CFS or ICF. Therefore, unlike most prior published studies, we did not find evidence of a gene expression biomarker for chronic fatiguing illness.

### Methodological Issue: genetic matching in gene expression studies

These results may hold a lesson for case-control gene expression studies in humans. There are certainly examples where transcriptomic studies have yielded results that have the potential to improve disease prognosis and management (e.g., breast cancer) [34]; however, gene expression studies have the potential to yield false positive findings if the ancestry of cases and controls are not appropriately matched. Genetic background is usually not taken into consideration although gene expression is can be both heritable and under strong genetic control [35]. Relatively low-resolution studies in immortalized PBLs suggest that hundreds of human genes are under relatively strong genetic control by common genetic variants (e.g., the single nucleotide polymorphism “rs407257” is strongly associated (p∼10^−66^) with the expression level of glutathione S-transferase theta 1, *GSTT1*) [36]. The genetic variant rs407257 is variable in human populations (allele frequencies of 0.72, 0.64, and 0.39 in African, East Asian, and European samples) [37]. If case and control subjects are not extremely well-matched for genetic background (including for location within Europe) highly significant differences could occur because of bias from inappropriate case-control matching. This concern is particularly important for studies of PBLs as genes whose expression is under strong genetic control [36] are highly enriched for genes expressed in lymphoid tissue and lymphocyte cell populations (analyses using DAVID [38], data not shown).

Use of discordant monozygotic twins represents the best control for genetic background currently possible in humans. Assuming identify at the DNA level and control for experimental bias, gene expression differences in discordant monozygotic twins can be cleanly attributed to disease state. It is reasonable to consider if use of discordant monozygotic twins represents “over-matching”. In comparisons of unrelated cases and controls, gene expression differences are an amalgam of disease state, the RNA-level impact of genetic loci causal to the trait, and the effects of case-control genetic mismatching (i.e., non-causal loci that differ in frequency between cases and controls and which have strong control on gene expression). Use of discordant monozygotic twins yields more interpretable results particularly as there are large numbers of non-causal loci under genetic control. We would also argue that a gene expression study is a poor way to identify genetic loci causal to a disease when an alternative study design (the genome-wide association study) has been so successful [39].

### Conclusions

We were unable to identify a biomarker for chronic fatiguing illness in the transcriptome of peripheral blood leukocytes suggesting that positive findings in prior studies may have resulted from experimental bias.

## Supporting Information

Table S1 Shown are results from pathway analyses using SAFE to investigate KEGG pathways, GO keywords (BP = biological process, CC = cellular component, and MF = molecular function), and PFAM protein family groupings. These all had an empirical p-value (from permutation) <0.005 and were composed of ≥10 transcripts.
(0.11 MB DOC)

Figure S1 Quantile-quantile (QQ) plots from the planned paired analyses contrasting monozygotic (MZ) twins affected with chronic fatiguing illness versus their unaffected co-twins. CFS = chronic fatigue syndrome, ICF = idiopathic chronic fatigue. The observed distribution of statistical results conform to chance expectations.
(0.11 MB DOC)
