# Supplementary material for: Gene Expression in Peripheral Blood Leukocytes in Monozygotic Twins Discordant for Chronic Fatigue: No Evidence of a Biomarker
Source: PLoS One. 2009 Jun 5;4(6):e5805. doi: 10.1371/journal.pone.0005805 (PMC2688030; doi:10.1371/journal.pone.0005805)
Supplement: Table S1 — Shown are results from pathway analyses using SAFE to investigate KEGG pathways, GO keywords (BP = biological process, CC = cellular component, and MF = molecular function), and PFAM protein family groupings. These all had an empirical p-value (from permutation) <0.005 and were composed of ≥10 transcripts. (0.11 MB DOC) [file pone.0005805.s001.doc]

| **Source** | **ID** | **Size** | **P empirical** | **Type** |
| --- | --- | --- | --- | --- |
| GO:BP | GO:0031497 | 192 | 0.0009 | chromatin assembly |
| GO:BP | GO:0006323 | 249 | 0.0018 | DNA packaging |
| GO:BP | GO:0043624 | 55 | 0.0022 | cellular protein complex disassembly |
| GO:BP | GO:0032984 | 60 | 0.0031 | macromolecular complex disassembly |
| GO:BP | GO:0006333 | 326 | 0.0036 | chromatin assembly or disassembly |
| GO:BP | GO:0043241 | 57 | 0.0037 | protein complex disassembly |
| GO:BP | GO:0006334 | 158 | 0.0038 | nucleosome assembly |
| GO:BP | GO:0031365 | 44 | 0.0038 | N-terminal protein amino acid modification |
| GO:BP | GO:0065004 | 325 | 0.0044 | protein-DNA complex assembly |
| GO:BP | GO:0016054 | 77 | 0.0046 | organic acid catabolic process |
| GO:BP | GO:0046395 | 77 | 0.0046 | carboxylic acid catabolic process |
| GO:BP | GO:0022411 | 93 | 0.0048 | cellular component disassembly |
| GO:CC | GO:0005852 | 27 | 0.0004 | eukaryotic translation initiation factor 3 complex |
| GO:CC | GO:0030894 | 34 | 0.0009 | replisome |
| GO:CC | GO:0043601 | 34 | 0.0009 | nuclear replisome |
| GO:CC | GO:0043596 | 40 | 0.0016 | nuclear replication fork |
| GO:MF | GO:0003857 | 15 | 0.0004 | 3-hydroxyacyl-CoA dehydrogenase activity |
| GO:MF | GO:0003988 | 12 | 0.0013 | acetyl-CoA C-acyltransferase activity |
| GO:MF | GO:0051087 | 39 | 0.0015 | chaperone binding |
| GO:MF | GO:0016790 | 239 | 0.0029 | thiolester hydrolase activity |
| GO:MF | GO:0008190 | 11 | 0.0036 | eukaryotic initiation factor 4E binding |
| GO:MF | GO:0050840 | 13 | 0.0040 | extracellular matrix binding |
| GO:MF | GO:0031072 | 150 | 0.0048 | heat shock protein binding |
| KEGG | KEGG:00280 | 115 | 0.0001 | Valine, leucine and isoleucine degradation |
| KEGG | KEGG:00062 | 22 | 0.0004 | Fatty acid biosynthesis (path 2) |
| KEGG | KEGG:00640 | 81 | 0.0006 | Propanoate metabolism |
| KEGG | KEGG:00071 | 115 | 0.0016 | Fatty acid metabolism |
| KEGG | KEGG:00380 | 143 | 0.0030 | Tryptophan metabolism |
| KEGG | KEGG:00530 | 56 | 0.0046 | Aminosugars metabolism |
| PFAM | PFAM:00156 | 20 | 0.0027 | Phosphoribosyl transferase domain |
| PFAM | PFAM:00675 | 16 | 0.0033 | Insulinase |
| PFAM | PFAM:00965 | 12 | 0.0033 | Tissue inhibitor of metalloproteinase |
| PFAM | PFAM:00725 | 14 | 0.0045 | 3-hydroxyacyl-CoA dehydrogenase, C-terminal domain |
| PFAM | PFAM:02737 | 14 | 0.0045 | 3-hydroxyacyl-CoA dehydrogenase, NAD binding domain |
