# Supplementary material for: Gene Expression in Peripheral Blood Leukocytes in Monozygotic Twins Discordant for Chronic Fatigue: No Evidence of a Biomarker
Source: PLoS One. 2009 Jun 5;4(6):e5805. doi: 10.1371/journal.pone.0005805 (PMC2688030; doi:10.1371/journal.pone.0005805)

(a) QQ plot for 44 pairs of MZ twins discordant for CFS or ICF.


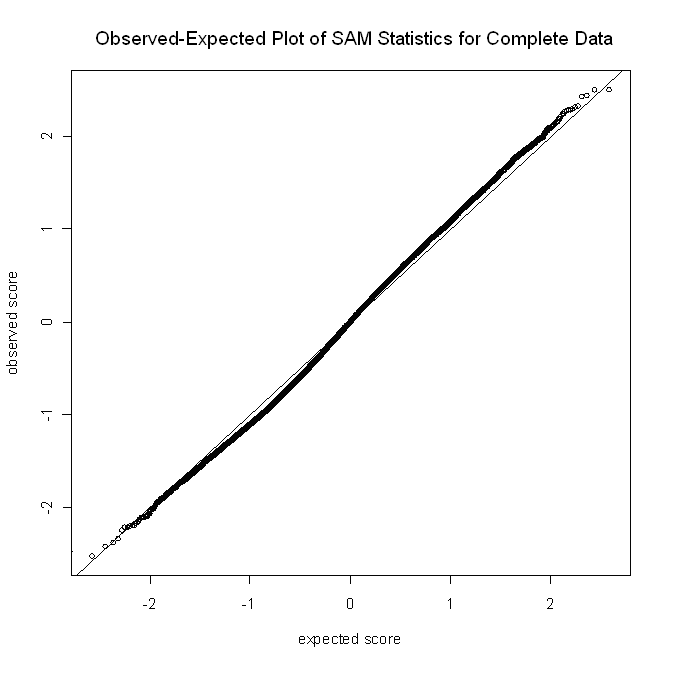


(b) QQ plot for 39 pairs of female MZ twins discordant for CFS or ICF.


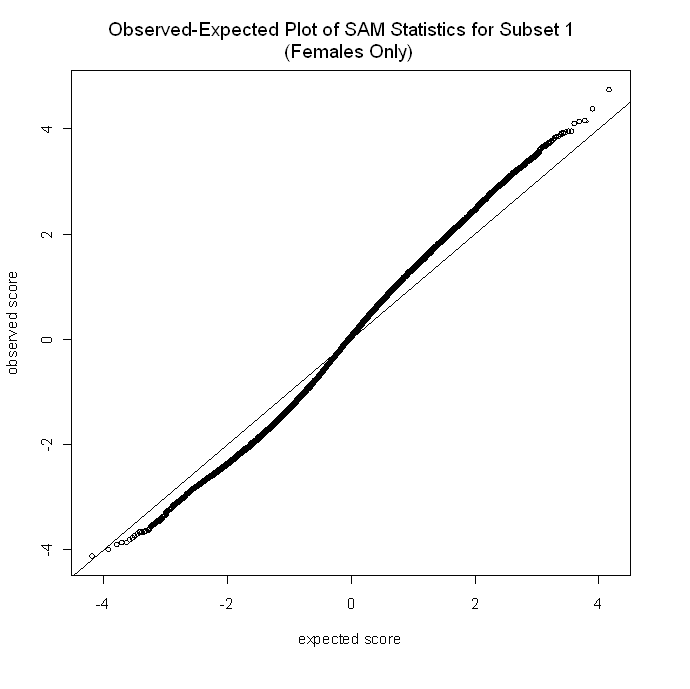


(c) 28 pairs of female MZ twins discordant for CFS.


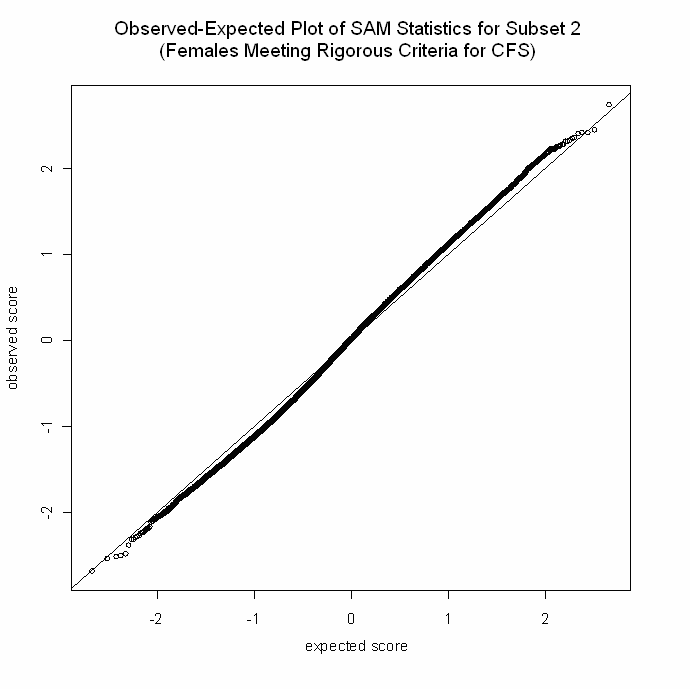

Supplement: Figure S1 — Quantile-quantile (QQ) plots from the planned paired analyses contrasting monozygotic (MZ) twins affected with chronic fatiguing illness versus their unaffected co-twins. CFS = chronic fatigue syndrome, ICF = idiopathic chronic fatigue. The observed distribution of statistical results conform to chance expectations. (0.11 MB DOC) [file pone.0005805.s002.doc]
